# Diagnosis of Asthma Based on Routine Blood Biomarkers Using Machine Learning

**DOI:** 10.1155/2020/8841002

**Published:** 2020-05-14

**Authors:** Jun Zhan, Wen Chen, Longsheng Cheng, Qiong Wang, Feifei Han, Yubao Cui

**Affiliations:** ^1^School of Economics and Management, Nanjing University of Science and Technology, Nanjing 210094, Jiangsu Province, China; ^2^Department of Clinical Laboratory, Wuxi People's Hospital Affiliated to Nanjing Medical University, Wuxi 214023, Jiangsu Province, China

## Abstract

Intelligent medical diagnosis has become common in the era of big data, although this technique has been applied to asthma only in limited contexts. Using routine blood biomarkers to identify asthma patients would make clinical diagnosis easier to implement and would enhance research of key asthma variables through data mining techniques. We used routine blood data from healthy individuals to construct a Mahalanobis space (MS). Then, we calculated Mahalanobis distances of the training routine blood data from 355 asthma patients and 1,480 healthy individuals to ensure the efficiency of MS. Orthogonal arrays and signal-to-noise ratios were used to optimize blood biomarker variables. Receiver operating characteristic (ROC) curve was used to determine the threshold value. Ultimately, we validated the system on 182 individuals based on the threshold value. Out of 35 patients with asthma, MTS correctly classified 94.15% of patients. In addition, 97.20% of 147 healthy individuals were correctly classified. The system isolated 7 routine blood biomarkers. Among these biomarkers, platelet distribution width, mean platelet volume, white blood cell count, eosinophil count, and lymphocyte ratio performed well in asthma diagnosis. In brief, MTS shows promise as an accurate method to identify asthma patients based on 7 vital blood biomarker variables and threshold determined by the ROC curve, thus offering the potential to simplify diagnostic complexity and optimize clinical efficiency.

## 1. Introduction

Asthma is a common chronic condition of the airways characterized by reversible airflow obstruction, airway hyper-responsiveness, and clinical symptoms that include wheezing, breathlessness, and chest tightness. Best estimates report that approximately 300 million people worldwide suffer from asthma, representing 4.3% of the global population [1]. In 2011, more than 26 million US adults reported asthma exacerbations, and $56 billion in economic burden was estimated to result from asthma [2]. According to data from the US Centers for Disease Control and Prevention, 3,615 people died in 2015 due to complications from asthma or about 1.1 in 100,000 individuals. Through 2015, 358 million people worldwide have had asthma, up from 183 million by 1990 [3]. Thus, asthma is a common global medical issue that remains challenging to address.

Intelligent asthma diagnosis is a trending topic in medical intelligent diagnosis, which is the use of artificial intelligence to diagnose medical conditions. Several studies have reported the diagnosis of asthma using data mining algorithms and methods applied to intelligent diagnosis, such as support vector machine (SVM) [4, 5] and neural networks [5–8]. Finkelstein and Wood used naïve Bayesian and SVM methods to successfully predict asthma accelerations on day eight with 80% accuracy in a population of 26 patients through home telemedicine [4]. Methods of deep neural networks deployed to classify morbid conditions as well as collect lung performance values indicate the possibility of training a deep neural network to predict asthma severity or the imminence of an asthma attack [6].

Similarly, Badnjevic and Cifrek applied a trained neural network and fuzzy rules to assist physicians in the analysis and interpretation of pulmonary function test results, successfully improving asthma detection, diagnosis, and treatment [7]. Using data mining in the diagnosis of asthma, Safdari et al. evaluated the sensitivity, specificity, and accuracy of the K-nearest neighbor, SVM, naive Bayes, artificial neural network, classification tree, CN2 algorithms, and similar techniques, all of which are based on 24 attributes [5]. SVM algorithms achieved the highest accuracy at 98.59%, with 98.6% sensitivity and specificity. In another study of asthma control in children, algorithms based on artificial neural networks and principal component analysis of lung function parameters and fractional exhaled nitric oxide correctly identified 99.0% of children with totally controlled asthma [8].

Currently, there is no gold standard for asthma diagnosis. Cell types involved in pathogenesis of bronchial asthma include T lymphocytes, eosinophils, basophils, mast cells, and bronchial epithelial cells. An association between peripheral blood eosinophilia and moderate-to-severe asthma has been well defined, and an elevated eosinophil level of at least 400 cells/*μ*L has been associated with greater use of healthcare resources via increased hospital admissions and costs [9]. Furthermore, high blood eosinophil count is a risk factor for future asthma exacerbations and excessive short-acting *β*-agonist use after adjustment for potential confounders in adults with persistent asthma [10]. In the course of multiple exacerbations of the disease, an increased number of neutrophils can be detected in peripheral blood. However, the role of neutrophils in the pathogenesis of bronchial asthma remains unclear. In addition, platelet counts and mean platelet volume (MPV) are higher in asthmatic children than control children with no evidence of allergic disease (i.e., asthma, allergic rhinitis, or eczema), and mean MPV during an asymptomatic period is higher in individuals with exacerbated asthma than in healthy controls [11].

Standardized criteria involving both assessment of risk factors and measurement of blood biomarkers that predict the risk of asthma exacerbation could provide more optimal treatment guidance and reduce healthcare costs. However, although complete blood counts are routinely ordered for asthma patients, they do not yet provide a clear indication of such biomarkers.

The Mahalanobis–Taguchi system (MTS) is a decision-making and pattern recognition system frequently used as a multidimensional system to integrate information to construct reference scales by creating individual measurement scales. This system is an organic combination of Mahalanobis distance (MD) and the Taguchi method. MD is a generalized distance that helps discriminate similarities between unknown and known sample datasets. The Taguchi method optimizes the system and evaluates the contribution of each variable [12]. The system focuses on orthogonal arrays (OAs) and signal-to-noise (SN) ratios to identify variables of importance, which form a basis to construct a reduced model of measurement scale. Selecting an optimal subset of the most important variables from the original variable set is essential to MTS [13, 14], which differs from other classification methods such as SVM and neural networks. MTS uses a single category sample to form a continuous measurement scale. Rather than direct experimentation, all training data sets are used to construct a classification model.

Recently, several researchers have used MTS for intelligent disease recognition with high accuracy [15, 16]. However, no research has used MTS for intelligent diagnosis of asthma. The purpose of this study was to apply MTS to asthma diagnosis based on assessment of routine blood data from healthy individuals and asthma patients. We sought to identify routine blood biomarkers that could indicate asthma and a reduced model construction of measurement scale. We also compared MTS results with other algorithms to determine which had best accuracy, sensitivity, and specificity. These results can be applied to asthma diagnosis decision systems.

## 2. Methods

### 2.1. Data Acquisition

We analyzed routine blood data from 355 asthma patients and 1,480 healthy individuals collected at Wuxi People's Hospital affiliated with Nanjing Medical University by laboratory personnel with medical and technical training. Samples included data from diagnosis of asthma patients and from physical examinations of healthy individuals. Asthma was diagnosed and classified according to the Global Initiative for Asthma 2015 Global Strategy for Asthma Management and Prevention [17]. Basic information about the study population is presented in Table 1. This study has been approved by the hospital ethics committee (KYLLH2018034), and all patients signed informed consent.

### 2.2. Preprocessing

Routine blood data were assessed to predict whether a blood sample was from an asthma patient or healthy control. Data preprocessing included the following steps.

#### 2.2.1. Handling Missing Data

A missing at random pattern was observed for the sample, with few incomplete data (with respect to the 22 variables examined). Three instances of a single missing variable value were removed from the analysis.

#### 2.2.2. Reducing Highly Correlated Variables

MTS is a quantitative analysis method. We found 22 initial routine blood variables (*X*_1_ ~ *X*_22_) that were highly correlated. It was necessary to use variable selection to avoid multicollinearity. Pearson correlation analysis was used with SPSS software to reduce model complexity using routine variables from healthy individuals. Nine groups of variables showed significant correlation >80% (Table 2). The final selected 14 variables for MTS were basophil count (BA#), eosinophil count (EO#), lymphocyte ratio (LY), lymphocyte count (LY#), mean corpuscular hemoglobin (MCH), mean corpuscular hemoglobin concentration (MCHC), monocyte ratio (MO), monocyte count (MO#), mean platelet volume (MPV), platelet distribution width (PDW), platelet count (PLT), red blood cell count (RBC), red blood cell distribution width (RDW), and white blood cell count (WBC).

### 2.3. Improved MTS Algorithm

We used MTS for data classification [18–25]. In MTS, Mahalanobis space (MS; reference group) is obtained using standardized variables of healthy or normal data. MS can be used to differentiate normal and abnormal. Once MS is established, the number of attributes is reduced using orthogonal arrays (OAs) and signal-to-noise (SN) ratios by evaluating the contribution of each attribute. Finally, unknown samples are identified by threshold value. More details of the MTS algorithm can be found in [14].

For the diagnosis of unknown samples, a precise threshold value is important. In the traditional MTS, quality loss function was proposed to determine threshold value by Dr. Taguchi. However, because it is too subjective to calculate, few scholars use it since it was proposed. Su et al. used Chebyshev's theorem to build a possibility threshold model called the “probabilistic thresholding method” (PTM) to determine the threshold value [26]. However, PTM ignored the number of false negative observations according to the rules.

In this article, receiver operating characteristic (ROC) curve was chosen to decide the threshold value. It has been widely applied to do medical diagnosis. We use MD of the normal and abnormal data in the training set to draw the ROC curve. On the basis of ROC curve rules, the point which makes sensitivity (Se) plus specificity (Sp) maximum is the best threshold value. Sensitivity is the probability that a test result will be positive when the disease is present (true positive rate). A sensitivity of 100% indicates correct detection of all disease patients. Specificity is a measure to identify negative cases of test data. A specificity of 100% indicates correct detection of all healthy individuals. Moreover, the area under the curve (AUC) was often used when estimating the classifier availability. Compared with the quality loss function, PTM, and exhaustive search method, ROC curve is more objective and visible.

**Figure 1:**
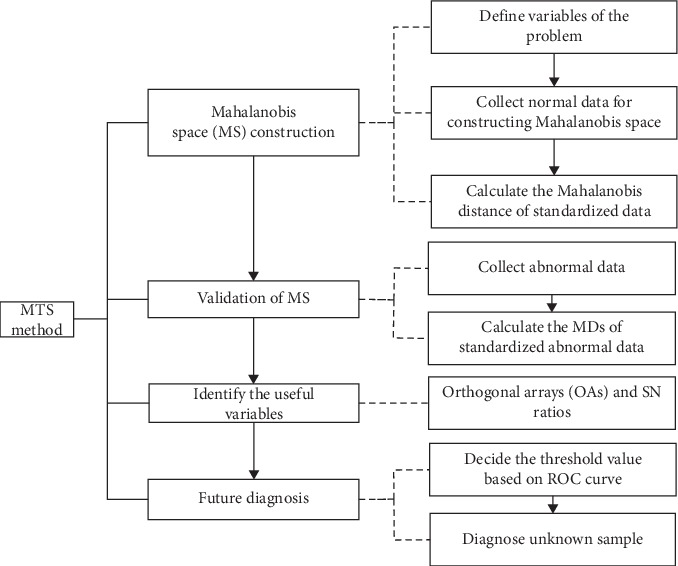
The flowchart of rolling bearing fault diagnosis.

The algorithm flowchart is shown in Figure 1.

## 3. Results

### 3.1. Improved MTS with Routine Blood Data

We used 10-fold cross-validation to research the dataset. For each loop, nine folds were used for training, and the remaining were used for testing data mining algorithms. Thus, there were 1,331 healthy individuals for normal training samples and 147 healthy individuals for testing samples. Also, there were 319 asthmatic patients for abnormal training samples and 35 asthmatic patients for testing samples. Implementation of the improved MTS is as follows.

**Figure 2:**
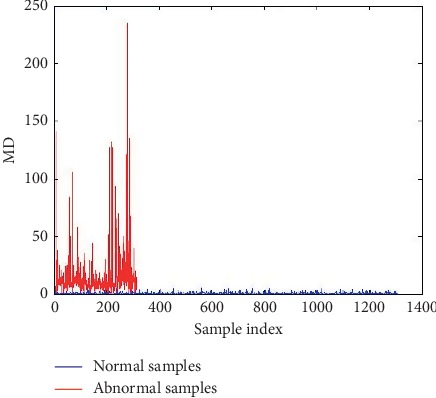
Mahalanobis distance (MD) for normal (healthy) and abnormal (asthma) samples.

In the first stage, MD of healthy samples was constructed using 14 variables. We find MDs of 106 datasets from 1,331 healthy datasets which were beyond the threshold ($T=\mu +2.66\bar{R}$ [27]). Then, we used 1,225 datasets to construct the MS. In the second MTS stage, calculation of abnormal (asthmatic) MD after constructing MS for the normal group was done. They were larger than normal, illustrating the classification ability of MD.  Figure 2 represents the MD of normal and abnormal data.

In the third stage of analysis, useful variables were selected by OAs and SN ratios. We used a L_16_ (2^15^) OA, a fractional factorial design that can accommodate up to 15 factors with 16 runs. We assigned the 14 variables to the first 14 OA columns, and the remaining columns were ignored. MD values were calculated for all asthma patients for the 14 variable combinations above indicated by OA rows. To obtain SN ratios, working averages were used as the values of SNR_*j*_, with *j* = 1, 2, ..., 16.  Table 3 presents L_16_ (2^14^) OAs and SN ratios. Gain in average value of the SN ratio was calculated for each variable.

**Figure 3:**
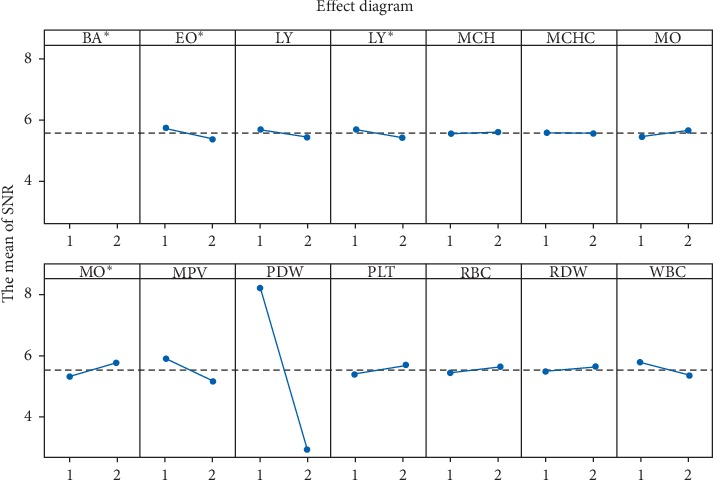
Mahalanobis space optimization results for selected variables using signal-to-noise ratio (SNR).

Figure 3 shows optimization results. Descending lines indicate $\bar{S/N{\text{ratio}}_{\text{level}1}}-\bar{S/N{\text{ratio}}_{\text{level}2}}$ > 0 and positive gains. Features *X*_4_ (EO#), *X*_7_ (LY), *X*_8_ (LY#), *X*_10_ (MCHC), *X*_14_ (MPV), *X*_18_ (PDW), and *X*_22_ (WBC) had positive gains and thus were selected to construct MS and calculate MD. SN ratio scores of 5.11 for PDW and 0.70 for MPV indicated that these variables were important for diagnosis. Rising lines indicate $\bar{S/N{\text{ratio}}_{\text{level}1}}-\bar{S/N{\text{ratio}}_{\text{level}2}}$ < 0 and negative gains. Because variables with negative gains did not significantly affect the system, they were neglected. After all insignificant variables were removed, MS and MD were recalculated for only 7 variables, reducing the number of variables to half.

**Figure 4:**
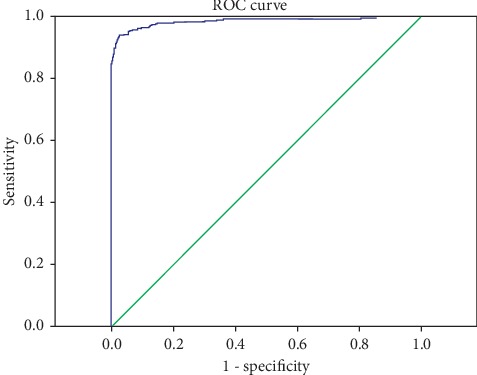
ROC curve.

With the model described above, selected useful variables were able to classify healthy and asthma cases. After that, the threshold value was calculated to distinguish between healthy and asthma samples. Draw up ROC curve (Figure 4) by software SPSS, the threshold value which maximize Se (0.937) plus Sp (0.974) is 1.911. If MD of one observation is large than 3.3673, the compound should be recognized as the asthma patient; otherwise, it is recognized as healthy people. The AUC 0.983 manifests this classifier is good and acceptable.

The correlation coefficient matrix, mean, and SD of healthy sample data with only 7 variables were used for the 182-sample testing set (containing healthy and asthma groups). The average Se was 94.15%, and the average Sp was 97.20%, indicating the method identified patients and healthy people with high accuracy.

### 3.2. MTS versus SVM

**Figure 5:**
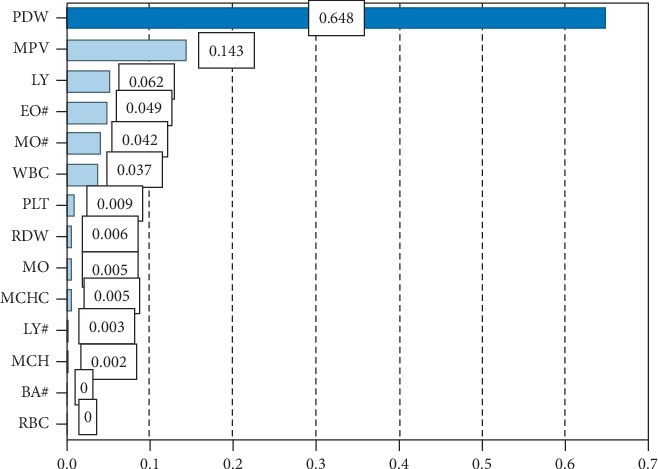
Variable importance scores from the support vector machine model.

SVM has high accuracy in classification, so we compared the performance of MTS with SVM. The SVM algorithm was calculated with Clementine software.  Figure 5 shows variable importance scores in SVM classification, with top scores for PDW (0.648) and MPV (0.143). Variable performance results based on SVM were consistent with MTS results. In addition, LY, EO#, MO#, and WBC also affected classification results. The accumulating contribution rate of these six variables was 97.1%. With reference to MTS, PDW, MPV, WBC, LY, and EO# performed well in asthma diagnosis.

Analysis of sensitivity and specificity of testing datasets under MTS with 7 variables, SVM with 14 variables, and SVM with 7 variables indicates that MTS performed better than SVM (Table 4). In addition, SVM with 14 variables had worse classification results than SVM with 7 variables. Both methods with 7 variables had good performance in specificity metrics.

## 4. Discussion

Our assessment of MTS to determine useful variables for predicting asthma diagnosis shows that MTS is a useful diagnostic and forecasting technique. It not only executes classification tasks but it also identifies important variables in a multivariate system. Compared to similar studies, the advantages of our approach can be summarized as follows:

1. MTS provides easier access to asthmatic diagnosis for patients by using routine blood test data. The algorithm can distinguish between asthmatics and healthy people.
2. MTS establishes a MS with data training as the reference space. Doctors only need to calculate the MD of unknown patients from the reference space to use software to diagnose if patients have asthma. Compared with other algorithms, such as SVM hyperplanes and neural network structures, MTS is easier to understand.
3. MTS provides a methodical way to identify asthma, reducing dimensionality of the diagnosis problem. It optimizes the reference space, removes redundant variables, and greatly reduces time complexity of the algorithm by OAs and SN ratios. This study shows good performance with PDW, MPV, WBC, EO#, LY#, LY, and MCHC variables. These key variables can provide clear guidance to doctors for asthma diagnosis. Doctors can use these 7 variables to diagnose patients by calculating MDs, thus simplifying diagnostic complexity and optimizing clinical efficiency.
4. MTS performed better than SVM in asthma diagnosis. Furthermore, with the onset of big data, MS can be built more completely, and thresholds will become more accurate. Therefore, MTS represents a new way to approach asthma diagnosis.

Some important works must be done to improve our findings. First, building a blood database of asthma patients and healthy controls would establish a complete reference space to more accurately identify asthma patients. Second, software should be developed and updated to facilitate asthma diagnosis using MTS. Third, the diagnostic process described here should be confirmed with patient samples of increasing asthma severity to construct another MTS that can identify asthma severity. In line with MTS theory, if a sample's MD is more distant from the reference space, the patient's asthma may be more severe. However, this study does not provide a specific scale or scope of reference MDs for asthma severity, although those could be determined with asthma patient data or by using multiclass MTS to identify diagnosis.

## 5. Conclusion

This study provides a clinical asthma diagnosis algorithm based on routine blood data that performs well in disease recognition. The algorithm discovered 7 variables of routine blood biomarker data that are vital to asthma diagnosis: PDW, MPV, WBC, EO#, LY#, LY, and MCHC. Further studies are required to extend this diagnostic to disease severity.

## Figures and Tables

**Table 1:** Basic characteristics of the study population.

Category	*N*	Age, years (*X* ± *S* *D*)	Sex (*n* (%))	
			M	F
Asthma	355	39.14 ± 22.60	175 (49.3%)	180 (50.7%)
Healthy	1,480	40.77 ± 12.71	763 (51.55%)	717 (48.45%)

**Table 2:** Pearson correlation of selected variables.

Variable A	Variable B	Pearson correlation	Reserved variable	Variable A	Variable B	Pearson correlation (%)	Reserved variable
BA	BA#	80.5	BA#	LY	NE	94.9	LY
EO	EO#	94.3	EO#	MCH	MCV	96.4	MCH
HCT	HGB	98.8	HGB	NE#	WBC	90.5	WBC
HCT	RBC	85.8	RBC	PCT	PLT	86.5	PCT
HGB	RBC	81.3	RBC				

**Table 3:** Orthogonal arrays (OAs) and signal-to-noise (SN) ratios for 14 variables.

No.	BA#	EO#	LY	LY#	MCH	MCHC	MO	MO#	MPV	PDW	PLT	RBC	RDW	WBC	SN ratio
1	1	1	1	1	1	1	1	1	1	1	1	1	1	1	8.29
2	1	1	1	1	1	1	1	2	2	2	2	2	2	2	3.36
3	1	1	1	2	2	2	2	1	1	1	1	2	2	2	8.38
4	1	1	1	2	2	2	2	2	2	2	2	1	1	1	3.10
5	1	2	2	1	1	2	2	1	1	2	2	1	1	2	2.94
6	1	2	2	1	1	2	2	2	2	1	1	2	2	1	7.87
7	1	2	2	2	2	1	1	1	1	2	2	2	2	1	2.98
8	1	2	2	2	2	1	1	2	2	1	1	1	1	2	7.16
9	2	1	2	1	2	1	2	1	2	1	2	1	2	1	8.40
10	2	1	2	1	2	1	2	2	1	2	1	2	1	2	3.54
11	2	1	2	2	1	2	1	1	2	1	2	2	1	2	7.34
12	2	1	2	2	1	2	1	2	1	2	1	1	2	1	3.70
13	2	2	1	1	2	2	1	1	2	2	1	1	2	2	2.10
14	2	2	1	1	2	2	1	2	1	1	2	2	1	1	9.36
15	2	2	1	2	1	1	2	1	2	2	1	2	1	1	2.72
16	2	2	1	2	1	1	2	2	1	1	2	1	2	2	8.50
$\bar{S/N{\text{ratio}}_{\text{level}1}}$	5.51	5.76	5.73	5.74	5.59	5.61	5.54	5.40	5.96	8.16	5.48	5.53	5.56	5.80	
$\bar{S/N{\text{ratio}}_{\text{level}2}}$	5.71	5.45	5.48	5.49	5.62	5.60	5.68	5.83	5.26	3.05	5.75	5.70	5.66	5.41	
Gain	−0.20	0.31	0.25	0.25	−0.03	0.01	−0.14	−0.43	0.70	5.11	−0.27	−0.17	−0.10	0.39	

**Table 4:** Sensitivity, specificity, and accuracy of algorithms.

	MTS with 7 variables (%)	SVM with 14 variables (%)	SVM with 7 variables (%)
Se	94.15	92.20	93.55
Sp	97.20	96.32	96.80

## Data Availability

The data used to support the findings of this study are available from the corresponding author upon request.
